# Reply to Losick, “Concerns about Continuing Claims that a Protein Complex Interacts with the Phosphorelay”

**DOI:** 10.1128/mBio.00154-20

**Published:** 2020-03-10

**Authors:** David Dubnau

**Affiliations:** a Public Health Research Institute, New Jersey Medical School, Rutgers University, Newark, New Jersey, USA

**Keywords:** bacterial development, Fe-S clusters, Ric proteins

## REPLY

Our recent paper in *mBio* (1) prompted Richard Losick to criticize our hypothesis for Ric protein function (2, 3). Although his concerns are not with this recent paper, R. Losick prefers a different hypothesis for Ric protein function (4, 5). However, our respective models are not mutually exclusive. There is no evidence showing that our model is wrong, and there is evidence supporting our model as well as the compatible one proposed by R. Losick.

Our starting point was with the observations that inactivation of *ricA* (*ymcA*), *ricF* (*ylbF*), or *ricT* (*yaaT*) prevented biofilm formation, genetic competence, and sporulation (6–8), all processes requiring the phosphorylation of Spo0A. The use of reporter constructs confirmed that the activation of Spo0A was dependent on the Ric proteins, while alleles of *spo0A* that suppress defects in the phosphorelay bypassed the *ric* requirements for transcription of Spo0A-P-dependent genes (2, 9). Similar results had already been independently reported for RicT (7). Importantly, a purified complex of the three Ric proteins stimulated the phosphorelay two- to threefold *in vitro* (2), so we proposed this direct action as the role of the Ric proteins.

The Losick lab then published papers proposing that these proteins were directly involved in RNA processing (4, 5). Since then we have acknowledged repeatedly that the Ric proteins are required *in vivo* for RNA processing events (1, 3, 9), and we have ourselves confirmed and extended this observation (1). Furthermore, we showed explicitly that the phosphorelay effect was only part of the Ric story, and we even proposed that the Losick hypothesis could explain the non-phosphorelay-related phenotypes (3, 9). In what follows we will maintain that neither model has been rigorously proven. Although neither our *in vivo* data nor R. Losick’s data prove that Ric proteins play direct roles in the phosphorelay or in RNA processing, in each case additional evidence has been claimed to support such direct roles. How convincing is this evidence? We will address this critically, first for our model and then for that of R. Losick.

Our demonstration that the Fe-S-containing Ric complex stimulates the phosphorelay *in vitro* (2, 3) is strong evidence that this is a direct role for the complex. R. Losick criticizes our experiments, claiming that “the stimulation experiments lacked the control of testing other proteins”. This criticism is mistaken. Four controls were reported (2, 3) as follows. In control 1, the addition of bovine serum albumen had no stimulatory effect. In control 2, the holo-complex was exposed to the atmosphere, leading to modification of the oxygen-sensitive Fe-S clusters, which prevented stimulation without inhibiting the phosphorelay. In control 3, the addition of glutathione *S*-transferase (GST) alone had no stimulatory effect, and in control 4, the addition of RicF-GST fusion protein, either alone or in complex with RicA and RicT, dramatically inhibited the phosphorelay reaction, supporting the notion that RicF interacted with one or another of the phosphorelay proteins.

R. Losick comments that “only a twofold stimulation” was observed, but he has himself characterized the phosphorelay as “a highly sensitive, self-reinforcing switch” (10), and a twofold effect might easily be significant *in vivo*. Our bacterial two-hybrid (B2H) analysis suggested a positive interaction of RicA with two phosphorelay proteins, Spo0F and Spo0B, but not with RicF and RicT (2). R. Losick comments critically that the B2H signal for these interactions was fourfold lower than the interaction of RicA with itself. But why would we expect the interaction of Spo0F with RicA to be stronger than that of RicA with itself? R. Losick is skeptical because he did not obtain evidence for RicA interaction with Spo0B. (He did not test Spo0F.) Perhaps the difference lies in the choice of different B2H systems with different fusion constructs. B2H data are suggestive, but we remain cautious in relying on such data, obtained with overexpressed proteins in a foreign host, possibly revealing low-affinity interactions that lack biological relevance. Indeed, it is generally appreciated that both false-positive results and false-negative results can result from two-hybrid screens, and B2H data should be confirmed by other means, a requirement that applies to our data and to R. Losick’s. The criticism that Spo0B and Spo0F resemble neither Rny nor one another is surprising; why must two proteins that each bind to a third exhibit similarities? R. Losick points out that our immunoprecipitation (IP) experiments did not reveal interactions of Ric proteins with Spo0F or Spo0B, but transient interactions might have been missed. Despite our B2H and *in vitro* phosphotransfer experiments, our model remains a plausible hypothesis buttressed by strong data that meets the test of Occam’s razor and awaits further confirmation. It is gratifying that R.L. at least admits our hypothesis to be “a formal possibility.”

The strongest, but still only suggestive, evidence for a direct involvement of Ric proteins in RNA metabolism derives from the reported interactions of Ric proteins with the RNase Rny. R. Losick provides several lines of evidence for these interactions. First is B2H data (4), reporting interactions of Rny with RicF and RicA, subject to the above-mentioned caveats. Second is his observation that Rny was detected by mass spectrometry when MBP-RicF was isolated after overexpression in Bacillus subtilis (4). RicA and RicT were not tested, and only a single biological replicate was reported for MBP-RicF. Our IP experiments with each of the three proteins suggested a possible interaction of Rny with RicT, but not with RicA or RicF (2). In these experiments, duplicate RicA and RicF IPs were performed, but only one sample was tested for RicT, and we made no claim for this interaction. Therefore, it appears that the detection of Rny-Ric interactions is variable at best and deserves more investigation. Finally, these experiments in our respective laboratories are difficult to compare because they were done using different constructs, different methods, in different strain backgrounds and with different growth conditions.

The Losick letter further proposes that the failure of both *rny* and *ric* mutants to cleave certain transcripts is evidence for the joint involvement of Rny and Ric in RNA processing. Again, this is not proof that the Ric proteins are directly involved in RNA processing. Finally, R. Losick points out that the “the Y-complex associates with the membrane in an RNase-dependent manner.” In fact, his paper showed this for RicT, not the complex; RicT is present in considerable excess over RicA and RicF (1). Because inactivation of *rny* affects many transcripts, in one study about 30% of them (11), this is a nonspecific finding that may be mediated indirectly. Despite these doubts, as we stated previously (1, 3, 9), we agree that the model advanced by DeLoughery et al. (4, 5) may be correct. In fact, the phosphorelay model is compatible with the Rny model because the Ric proteins may play more than one role. Examples abound of “moonlighting” proteins that serve more than a single function (see http://www.moonlightingproteins.org/). Germane examples are the glycolytic enzymes enolase and phosphofructokinase, which are also found in bacterial and yeast RNA degradosomes.

The Ric system is still poorly understood. Although the three proteins form a 1:1:1 complex, RicT is an order of magnitude more abundant in B. subtilis than RicA and RicF, and we have shown that RicT is soluble as a monomer, as is a homodimer of RicA and a heterotetramer of RicA and RicF (1). These forms may play independent roles *in vivo* (1). Interestingly, the *Clostridiales* carry genes that encode RicT, but not RicA or RicF. The presence of the Fe-S clusters is a further, unexplained embellishment of this intriguing system. We agree with R. Losick’s implied suggestion that we should interpret our own data cautiously, and we urge him to do likewise. We respectfully suggest that R. Losick demonstrate interactions of purified Rny with Ric proteins, perhaps even after reconstituting the Fe-S clusters under anaerobic conditions.

The bottom line is this: because no strong biochemical evidence showing that the Ric complex is directly involved in RNA processing exists, and we have no definitive mechanistic evidence for the interactions of Ric and phosphorelay proteins, we are faced with a symmetrical situation. Neither model has been proven, and in fact, both may be correct, as we have suggested (1, 3, 9). It is even conceivable that both models are wrong; perhaps the *in vivo* effects of *ric* deletions on the phosphorelay and on RNA maturation are indirect.

## References

[1] Adusei-Danso F, Khaja FT, DeSantis M, Jeffrey PD, Dubnau E, Demeler B, Neiditch MB, Dubnau D 2019 Structure-function studies of the Bacillus subtilis Ric proteins identify the Fe-S cluster-ligating residues and their roles in development and RNA processing. mBio 10:e01841-19. doi:10.1128/mBio.01841-19. 31530674 PMC6751060

[2] Carabetta VJ, Tanner AW, Greco TM, Defrancesco M, Cristea IM, Dubnau D 2013 A complex of YlbF, YmcA and YaaT regulates sporulation, competence and biofilm formation by accelerating the phosphorylation of Spo0A. Mol Microbiol 88:283–300. doi:10.1111/mmi.12186. 23490197 PMC3781937

[3] Tanner AW, Carabetta VJ, Martinie RJ, Mashruwala AA, Boyd JM, Krebs C, Dubnau D 2017 The RicAFT (YmcA-YlbF-YaaT) complex carries two [4Fe-4S]^2+^ clusters and may respond to redox changes. Mol Microbiol 104:837–850. doi:10.1111/mmi.13667. 28295778 PMC5444954

[4] DeLoughery A, Dengler V, Chai Y, Losick R 2016 Biofilm formation by *Bacillus subtilis* requires an endoribonuclease-containing multisubunit complex that controls mRNA levels for the matrix gene repressor SinR. Mol Microbiol 99:425–437. doi:10.1111/mmi.13240. 26434553 PMC4989519

[5] DeLoughery A, Lalanne JB, Losick R, Li GW 2018 Maturation of polycistronic mRNAs by the endoribonuclease RNase Y and its associated Y-complex in Bacillus subtilis. Proc Natl Acad Sci U S A 115:E5585–E5594. doi:10.1073/pnas.1803283115. 29794222 PMC6004469

[6] Branda SS, Gonzalez-Pastor JE, Dervyn E, Ehrlich SD, Losick R, Kolter R 2004 Genes involved in formation of structured multicellular communities by *Bacillus subtilis*. J Bacteriol 186:3970–3979. doi:10.1128/JB.186.12.3970-3979.2004. 15175311 PMC419949

[7] Hosoya S, Asai K, Ogasawara N, Takeuchi M, Sato T 2002 Mutation in *yaaT* leads to significant inhibition of phosphorelay during sporulation in *Bacillus subtilis*. J Bacteriol 184:5545–5553. doi:10.1128/jb.184.20.5545-5553.2002. 12270811 PMC139598

[8] Tortosa P, Albano M, Dubnau D 2000 Characterization of *ylbF*, a new gene involved in competence development and sporulation in *Bacillus subtilis*. Mol Microbiol 35:1110–1119. doi:10.1046/j.1365-2958.2000.01779.x. 10712692

[9] Dubnau EJ, Carabetta VJ, Tanner AW, Miras M, Diethmaier C, Dubnau D 2016 A protein complex supports the production of Spo0A-P and plays additional roles for biofilms and the K-state in Bacillus subtilis. Mol Microbiol 101:606–624. doi:10.1111/mmi.13411. 27501195 PMC4978174

[10] Chastanet A, Losick R 2011 Just-in-time control of Spo0A synthesis in *Bacillus subtilis* by multiple regulatory mechanisms. J Bacteriol 193:6366–6374. doi:10.1128/JB.06057-11. 21949067 PMC3209201

[11] Laalami S, Bessieres P, Rocca A, Zig L, Nicolas P, Putzer H 2013 *Bacillus subtilis* RNase Y activity in vivo analysed by tiling microarrays. PLoS One 8:e54062. doi:10.1371/journal.pone.0054062. 23326572 PMC3542257

